# Comparison of palpation and ultrasonography findings in supraspinous ligaments of horses

**DOI:** 10.3389/fvets.2026.1843226

**Published:** 2026-06-15

**Authors:** Maija Finnholm, Tytti Niemelä, Noora Eerikäinen, Jouni Junnila, Heli K. Hyytiäinen

**Affiliations:** 1 Department of Equine and Small Animal Medicine, Faculty of Veterinary Medicine, University of Helsinki, Helsinki, Finland; 2 Nevet, Mäntyharju, Finland; 3 EstiMates Oy, Turku, Finland

**Keywords:** back pain, desmitis, equine, reliability, validity

## Abstract

**Introduction:**

The purpose of this study was to evaluate the utility of palpation as a diagnostic tool for supraspinous ligament injuries in horses. Diagnosis of SSL injuries typically relies on a combination of clinical examination and ultrasonographic findings. To the authors’ knowledge, no previous studies have specifically investigated palpation as a diagnostic measure for these injuries.

**Methods:**

This retrospective study analyzed data from 126 horses resented to the University Veterinary Teaching Hospital for back pathology. Inclusion criteria required horses to have a documented descriptive palpatory finding of the dorsal midline and a corresponding ultrasonographic examination of the SSL performed during the same visit. Palpable findings, including swelling, pain, temperature differences, and other abnormalities, were recorded. The location of ultrasonographic findings was categorized as either the thoracic or lumbar spine. Sensitivities, specificities, positive predictive values (PPV), and negative predictive values (NPV) were calculated and analyzed.

**Results:**

The study concluded that palpation is a highly sensitive but less specific method for assessing signs associated with supraspinous ligament pathology. It was found to be particularly useful for detecting swelling in the supraspinous ligament area of the thoracic spine.

**Discussion:**

As a sensitive method, a positive palpatory finding can indicate the possibility of SSL injury and warrants further ultrasonographic investigation.

## 1 Introduction

Back pain is a common finding in horses (1, 2). However, reaching a definitive diagnosis of the cause can be a challenge. Clinical signs vary widely, with classical behavioral changes like reluctancy to move, bucking, or poor performance being the most common complaints (1). Primary back pain arises either from soft tissue injuries in the ligaments, tendons, or muscles supporting the spine or from bony spinal pathologies. Secondary or compensatory back pain can arise from locations outside the spine; for example, hindlimb lameness may affect thoracolumbar movement (1), thus causing dysfunction to the area.

One source of primary back pain is injury to the supraspinous ligament (SSL) (3, 4). The SSL is a strong cord of fibrous tissue located in the midline of the back, directly under the skin (5). It lies dorsal to the thoracic and lumbar spinous processes, where it stabilizes the thoracolumbar vertebrae (6). The etiology of SSL injuries is variable; possible causes can include direct trauma, saddle compression, injury in association with dorsal spinous processes, and overstretching due to exercise (5).

The diagnosis of SSL lesions is based on history, clinical examination including palpation of the back, and exclusion of other diseases; the diagnosis is confirmed with ultrasonography (4, 6). As in other ligament injuries, SSL lesions are characterized by hypoechoic areas, remodeling of fibers, and local thickening of the ligament in ultrasonography (3). Ultrasonographic images of the ligament are not always aligned with the clinical signs of the horse, so there might be abnormalities in echogenicity of the SSL in horses with no clinical findings or history of back pain (6, 7). Nevertheless, ultrasonography is still considered the gold standard of soft tissue imaging, especially of SSL injuries, in horses (3, 6).

Manual palpation is the ability to assess the integrity of superficial anatomical structures using one’s hands, and it remains one of the most important clinical examination methods to diagnose back pain in horses (8, 9). The SSL can be felt by applying various levels of pressure to the midline of the back, and injury manifests as localized swelling, temperature difference, or pain reaction (10, 11). SSL is densely innervated with sensory fibers and is thus overtly pain sensitive (12). In humans, despite palpation being a widely used assessment method, there is conflicting information regarding its use as a diagnostic method (13, 14). In a study in humans, the reliability of manual palpation tests in assessment of lower back patients varied greatly; static soft tissue palpation seemed to be more reliable than palpation of bony structures (13). Physiotherapists who routinely use palpation in their daily work detect changes in skin temperature more accurately than a layperson. Experienced practitioners can detect temperature differences of as little as one degree Celsius in the skin, this being precise enough to detect mild superficial inflammation in the skin (15).

In equine practice, manual palpation as a diagnostic method has been assessed and objectified by using pressure algometers (16, 17). Both palpation and algometer measurements have been utilized when assessing muscle-derived pain and nociceptive threshold. Based on comparison of these methods, palpation has been shown to be a repeatable assessment tool in horses (16, 18–20). Palpatory findings, such as muscle spasms and asymmetry, have been demonstrated to be predictive in diagnosing hindlimb fractures (21). However, the reliability and validity of equine SSL palpation as a diagnostic tool have not, to the authors’ knowledge, been studied previously. If shown to be valid, use of palpation might provide new avenues for SSL assessment. For example, each re-assessment of the injury during its rehabilitation period might not always require hair clipping and ultrasonography but could be assessed by palpation. Moreover, palpatory findings – even if incidental – might provide a trustworthy indication for further examination, when present in the SSL area.

We hypothesize that, compared with ultrasonography, palpation is a sensitive and specific method for detecting lesions in the SSL. Further, we hypothesize that especially a positive pain reaction in palpation will indicate a lesion and correlate to findings suggestive of SSL lesion.

## 2 Materials and methods

### 2.1 Horses

This retrospective study included horses examined for suspected back pathology at the University of Helsinki Veterinary Teaching Hospital over a 13-year period (June 2006 to July 2019). The sample comprised both referral and first opinion cases. Signalment of each included animal was recorded: age, breed, sex, and bodyweight. Data on horses’ history, clinical signs, treatments, details of findings in ultrasonographic examination, and diagnosis were collected from the hospital’s electronic patient records.

#### 2.1.1 Ethics approval

No ethical approval was needed at the time of the study, as all data were collected retrospectively in compliance with current data protection recommendations.

### 2.2 Method

#### 2.2.1 Data collection

To find information on the medical records of horses with suspected back pathology, the University of Helsinki Veterinary Teaching Hospital database was reviewed (Provet Net, Finnish Net Solutions Oy, Finland). Keywords used in the search were palpation, ultrasound, examination, imaging, thoracolumbar supraspinous ligament, thoracolumbar supraspinous ligament injuries, thoracic spine, lumbar spine, lumbar area, back, back pain, backpain, back muscles, back ligament, supraspinous ligament, midline, spinous process, epaxial, longissimus, and saddle area. Both English and Finnish (native language) words were used where applicable. Inclusion criteria for the study were a descriptive palpatory finding of the dorsal midline and a documented ultrasonographic exam of the SSL of the back on the same visit. The number of visits was recorded; some horses visited the hospital once and others had repeated visits. All visits with palpation and ultrasonographic examination were included and analyzed separately.

#### 2.2.2 Palpation of the back

Palpation was performed by a veterinarian (*n* = 9) and a physiotherapist (*n* = 4). All nine veterinarians were experienced, Finnish national specialists or diplomates of the European College of Veterinary Surgeons, practicing mainly equine orthopedics. All had at least 2 years of experience in diagnosing and treating equine back pain at the time of their contribution to this study. Four experienced physiotherapists were all specialized in veterinary physiotherapy. A horse could be examined by only a veterinarian or by two professionals (physiotherapist and veterinarian) working together. In the latter case, the physiotherapist performed the palpation and the veterinarian the ultrasonography.

Each horse’s manual palpation findings in the dorsal midline that could indicate a lesion in the SSL were recorded as follows: subcutaneous or ligamentous swelling “present” or “absent,” palpatory pain reaction “present” or “absent,” temperature difference “present” or “absent,” and other findings such as lumps or thickening of the SSL “present” or “absent.” A palpation finding was considered to be present if there was at least one of these following noted: swelling, pain reaction, temperature difference, or lumps or thickening. No grading of the palpatory finding was available due to its being subjective data collected retrospectively. For analysis purposes, the anatomical localization of the palpation findings was grouped into two areas: thoracic spine, comprising the first thoracic vertebra (T1) to the eighteenth thoracic vertebra (T18), and/or lumbar spine, comprising the first lumbar vertebra (L1) to the sixth lumbar vertebra (L6). Pain was identified through clinicians’ estimation of tissue-level reaction of behavioral responses from the horse. Obvious behavioral responses to palpation might include fasciculation, tensing muscles, extension of the thoracolumbar spine, moving away from the handler, or even pulling the ears back and tail swishing (22).

#### 2.2.3 Ultrasonography of the back

If a horse was suspected of SSL injury due to clinical signs or if there was a palpatory finding in the midline of the back, an ultrasonography of the SSL was performed. After palpation, prior to the ultrasonography, all horses were clipped over the midline of the back and aqueous contact gel and/or alcohol was used. A linear transducer of 9 MHz was used (GE Healthcare LogiQ P5). Longitudinal and cross-sectional views were obtained of the SSL. The ultrasonography findings of the SSL were categorized as “no injury,” “anechoic,” “hypoechoic,” “anechoic and hypoechoic”, “hyperechoic”, “unorganized fibers”, “thickening”, or “undefined desmitis.” Possible swelling in the ligament was recorded as “present” or “absent.” The location of ultrasonographic findings was categorized similarly to the above-described palpation findings: to the thoracic or lumbar spine.

### 2.3 Statistical analysis

Data were recorded in Excel, version 2,208 (Microsoft Inc., USA). All statistical analyses were done using SAS System for Windows, version 9.4 (SAS Institute Inc., USA). Frequency tables and descriptive statistics were calculated for horse demographics (sex, breed, age, and weight).

In addition to overall palpation findings, swelling and pain were analyzed separately. The category other findings in palpation (abnormal lumps or thickening of the SSL) was not analyzed as a separate finding, as it was so limited in number that statistics could not be performed. This category was also ignored in the analysis by location (thoracic or lumbar), as it was non-specific for SSL injuries and not the primary interest of this study.

Agreement of findings suggestive of SSL lesion between palpation and ultrasonography was evaluated (considering ultrasonography as the gold standard) by calculating sensitivities, specificities, positive predictive values, and negative predictive values with 95% confidence intervals (CIs).

## 3 Results

This study included data from 126 examinations of 101 horses (45 mares, 52 geldings, 4 stallions) assessed for back pain. Most of the patients (76.2%) were warmbloods (*n* = 77). Other breed categories were ponies (*n* = 8), Standardbreds (*n* = 5), Finnhorses (*n* = 3), Arabian horses (*n* = 3), crossbreeds (*n* = 3), and a Friesian horse (*n* = 1). The median age of horses was 9.5 years (range 3–21 years). The bodyweight was recorded in 34 of 101 cases. The median weight of horses, where it was recorded, was 538 kg (range 380–614 kg).

Sensitivity, i.e., the ability of a test to correctly identify horses with the disease (true positive rate), is considered good when values range from 0.75 to 0.9. Values between 0.5 and 0.75 are considered moderate and below 0.5 poor (23). In this study, palpatory findings had a sensitivity ranging from 58.3% to 85.7%. Overall, sensitivity of all palpation findings in all locations was good (82.4%). Specificity, i.e., the test’s ability to identify horses without the disease (true negative rate), is considered good for values ranging from 0.75 to 0.9, moderate for values between 0.5 and 0.75, and poor for values below 0.5 (23). In this study, specificity of palpation ranged from 42.9% to 87.2%. Palpation findings in all locations of the SSL had a poor specificity (42.9%). For a total of 126 horses, 95 examinations had a documented palpatory finding somewhere in the SSL, whereas 91 examinations had an ultrasonography finding suggestive of SSL lesion. The SSL examination revealed a finding in approximately 60% of the examinations in both palpation and ultrasonography (Table 1). Positive predictive value estimates the likelihood that a patient who is test-positive will have the disease. Similarly, negative predictive value estimates the likelihood that a patient who is test-negative is disease-free (23). Palpation in all locations had a positive predictive value of 78.9%, which is considered good, and a negative predictive value of 48.4%, which is considered poor. Of the horses, 13% had ultrasonographic findings suggestive of a lesion without palpatory findings in the SSL (Table 1).

**Table 1:** Cross-tabulation table with palpatory and ultrasonographic findings in the supraspinous ligament.

			Ultrasonography		
			Yes	No	Total
Palpation	All findings	Yes	59.5% (*n* = 75)	15.9% (*n* = 20)	75.4% (*n* = 95)
		No	12.7% (*n* = 16)	11.9% (*n* = 15)	24.6% (*n* = 31)
		Total	72.2% (*n* = 91)	27.8% (*n* = 35)	100% (*n* = 126)
	Swelling	Yes	9.5% (*n* = 12)	11.1% (*n* = 14)	21.1% (*n* = 26)
		No	1.6% (*n* = 2)	75.4% (*n* = 95)	78.9% (*n* = 97)
		Total	11.4% (*n* = 14)	88.6% (*n* = 109)	100% (*n* = 123)
	Pain	Yes	50% (*n* = 63)	12.7% (*n* = 16)	62.7% (*n* = 79)
		No	22% (*n* = 28)	15% (*n* = 19)	37.3% (*n* = 47)
		Total	72.2% (*n* = 91)	27.6% (*n* = 35)	100% (*n* = 126)
	Thoracic spine	Yes	53.2% (*n* = 67)	13.5% (*n* = 17)	66.7% (*n* = 84)
		No	16.7% (*n* = 21)	16.7% (*n* = 21)	33.3% (*n* = 42)
		Total	69.8% (n = 88)	30.2% (*n* = 38)	100% (*n* = 126)
	Lumbar spine	Yes	5.6% (*n* = 7)	12.7% (*n* = 16)	18.3% (*n* = 23)
		No	4.0% (*n* = 5)	77.8% (*n* = 98)	81.8% (*n* = 103)
		Total	9.5% (*n* = 12)	90.5% (*n* = 114)	100% (*n* = 126)

*n*, number of horses.

When looking at different anatomical locations, palpation findings in the thoracic spinal area had good sensitivity (76.1%), but specificity was considered poor (55.3%) (Table 2). Thoracic spinal palpatory findings were documented in a total of 84 examinations. Of examinations of the thoracic region, 53% had both palpation and ultrasonographic findings. The positive predictive value of palpatory findings in the thoracic spine was considered good (79.8%), while the negative predictive value was considered poor (50%).

**Table 2:** Sensitivity, specificity, positive predictive value, and negative predictive value of SSL palpation with 95% confidence intervals compared with gold standard ultrasonography.

Palpatory finding	Sensitivity (95% CI)	Specificity (95% CI)	PPV (95% CI)	NPV (95% CI)
All locations	82.4 (73.0–89.6)	42.9 (26.3–60.6)	78.9 (69.4–86.6)	48.4 (30.2–66.9)
Thoracic spine	76.1 (65.9–84.6)	55.3 (38.3–71.4)	79.8 (69.6–87.7)	50.0 (34.2–65.8)
Lumbar spine	58.3 (27.7–84.8)	86.0 (78.2–91.8)	30.4 (13.2–52.9)	95.1 (89.0–98.4)
Swelling	85.7 (57.2–98.2)	87.2 (79.4–92.8)	46.2 (26.6–66.6)	97.9 (92.7–99.7)
Pain	69.2 (58.7–78.5)	54.3 (36.6–71.2)	79.7 (69.2–88)	40.4 (26.4–55.7)

Cl, confidence interval; PPV, positive predictive value; NPV, negative predictive value.

Palpation findings in the lumbar spine had poor sensitivity (58.3%) (Table 2). Specificity in the lumbar spinal area was good (86%). Lumbar palpatory findings were present in only 23 of the 126 examinations. Only 5.6% of examinations had findings in the lumbar spine in both ultrasonography and palpation. The positive predictive value of palpatory findings in the lumbar spine was considered poor (30.4%), while the negative predictive value was high (95.1%).

Swelling in relationship to ultrasonography had the highest sensitivity (85.7%) with good specificity (87.2%). Palpable swelling was present in 27 of the 123 examinations. In the data collection phase, three examinations lacked information about swelling in ultrasonography and were excluded from the calculations. Swelling was documented in both palpation and ultrasonography in 9.5% of the examinations, and 11% of the examinations had palpatory findings but no ultrasonography findings in the SSL (Table 1). Only two examinations had no palpatory swelling but had ultrasonographic swelling (Table 1). Positive predictive value was considered poor (46.3%). Negative predictive value was 97.9%, which is high (23). Other findings were so limited in number that statistics could not be performed.

Pain in relationship to ultrasonography had moderate sensitivity (69.2%) and specificity (54.3%). Pain was documented in 79 of 126 horses. Pain in palpation and ultrasonographic findings suggestive of SSL lesions were documented in 50% of horses, and 12.7% of the examinations had palpatory findings but no ultrasonography findings in the SSL (Table 1). For palpation pain to be an indication of lesion in the SSL positive predictive value was considered good (79.7%). Negative predictive value was 40.4%, which is considered poor.

## 4 Discussion

Our hypothesis was that palpation, relative to ultrasonography, would be a sensitive and specific method for detecting lesions in the SSL in the back of the horse. This hypothesis was partly confirmed, with palpation findings in the SSL revealing good sensitivity (82.4%) but poor (42.9%) specificity for indicating lesions in the SSL compared with the gold standard ultrasonography in a sample of 126 equine back examinations.

A good sensitivity rate leads to more horses with SSL injuries being diagnosed in clinical practice. However, some horses are falsely identified as having the pathology and might be subjected to unnecessary investigations. Nevertheless, as the investigations are minimally invasive (ultrasonography), the main consequence would be hair clipping and some financial cost to the owner. Moreover, although palpation alone is not necessarily a sufficient diagnostic method, it might be useful as a follow-up method for the healing SSL. A diagnostic compromise with the most optimal test is chosen since the values are often but not always relatively inversely related; as sensitivity increases, specificity tends to decrease (24).

Swelling was the palpatory finding with the highest sensitivity (85.7%) and specificity (87.2%). Focal swelling, i.e., build-up of a larger amount of fluid due to acute inflammation, is described in the literature as one sign of acute SSL lesion (5). SSL lesion is seen as tissue enlargement or hypoechoic lesion in ultrasonography (5). In some horses, the SSL lesions disappear completely in ultrasonography when healed, while in others the appearance of the SSL never returns to normal (5). Ultrasonographic lesions as hyperechogenicity in the SSL have been shown to be visible long after injury, but how commonly these and others lesions present as swelling are seen afterwards is unknown. Further research of SL ultrasonographic changes and their grading in horses is needed. According to our results, palpable swelling was one indicator for SSL lesions. Thus, when suspicious of SSL injury the dorsal midline of the horse should be palpated bearing that in mind specifically. Swelling together with other palpatory findings in the SSL (pain reaction, temperature difference, and other findings as lumps or thickening) were all analyzed as one finding. Swelling was only present in a small number of horses. This brings uncertainty to how significant each of these individual palpatory findings is in relationship to SSL ultrasonography and clinical disease. When pooled, these findings showed good sensitivity but poor specificity.

One of the overall palpation findings in the SSL was palpable pain, with a moderate sensitivity (69.2%) to indicate lesion in the SSL. The SSL is densely innervated with nerve fibers and ultrasonographic findings suggestive of SSL lesion of the ligament has, in previous studies, been associated with pain reaction (1, 4, 12). The moderate specificity rate (54.3%) in palpable pain could be linked to the difficulty in diagnosing pain in horses; distinguishing painful palpable reactions from normal reactions is challenging (8). Pain recognition is highly individual and sometimes inconsistent. For veterinarians working with cattle, there are several contributing factors affecting sensitivity to recognize pain, including empathy, level of education, and previous work experience of the examiner (25). Horses have individual severity of injuries as well as sensation and signs of pain; some horses can show palpable reactions due to earlier experiences and learning of early avoidance and others can be hypersensitive on back palpation (1, 2, 26). Especially in the acute phase of inflammation, SSL is more painful. Pain reactions could help distinguish acute injuries from chronic injuries (5). Moreover, the back pain may arise from other structures of the back than the SSL, and these underlying pathologies may not always be identified (1). In a large study of horses with thoracolumbar complaint, 38.8% of lesions were shown to be soft tissue injuries, such as ligament or muscle-related injuries, but many other injuries exist, and there can be multiple sites of pathology at the same time (1). Overriding dorsal spinous processes are considered one of the most common causes of back pain in horses (1). The SSL stabilizes the thoracolumbar vertebrae and spinous processes, and these osseus structures are better diagnosed with radiography (1).

An increase in skin temperature was included in the overall palpation findings, but its specificity or sensitivity as an indicator of SSL lesion was not analyzed. Heat is one of the earliest signs of inflammation and should be felt in palpation by experienced clinicians (15). However, in a more chronic stage of the SSL injury, as acute signs decrease, this might not be present or could be missed in palpation (9).

The final finding that was assessed in the overall palpation findings was other findings as lumps or thickening of the SSL. The SSL lies directly under the skin, and in some horses thick adipose tissue might be mistaken for ligament thickening (5). Thickening of the SSL could also be due to fibrosis in a more chronic condition or even a healed lesion with less/no clinical relevance. This study did not exclude re-examinations and potentially more chronic lesions, and acute and chronic lesions were not distinguished retrospectively. Moreover, clinical relevance was not considered. Defining the age of the lesion in clinical setting even when utilizing all available assessment methods is challenging at best. The definition of the age of the lesion or the clinical relevance of it was not attempted due to the uncertainty in diagnosis. These could, however, be considered and explored in future studies. Presumably, acute SSL injuries might be easier to find, as it could be expected that acute injuries have more inflammation, and thus, swelling, pain, and heat would be present, which were found to be the most sensitive of findings. Chronic injuries could be expected to include fibrous tissue changes, causing scarring, i.e., lumps and bumps. The usefulness of palpation in monitoring SSL healing is unknown. Palpation might be a useful tool in monitoring SSL healing in horses; as local signs decrease in palpation, the exercise schedule of the horse could gradually be increased (5). Palpable findings in the thoracic region had a higher sensitivity (76.1%) than in the lumbar area (58.3%). Specificity, on the other hand, was higher in the lumbar area (86%) than in the thoracic region (55.3%). This is clinically relevant, as according to previous literature, injuries to SSL can occur in any region, but the most common location is between T15 and L3, i.e., the caudal thoracic area (5, 20). Therefore, our finding that injuries in the thoracic spine, the more common anatomic location for injury, are better detected than those in the lumbar spine is a positive one.

Based on our results, palpation of the SSL cannot replace ultrasonography as a diagnostic method. Ultrasonography gives real-time detailed information on SSL structure. However, when back pathology is suspected, palpation with its good sensitivity can be a useful indicator of SSL lesions and support the ultrasonographic evaluation and proper aftercare of horses. It can also be used as a screening method for SSL injuries. Moreover, palpation supports the ultrasonography results as part of the diagnosis-making process by enabling pain assessment, which diagnostic imaging cannot quantify.

There are several limitations to the study. Retrospectively reviewed data and a long study period led to the data being based on several clinicians’ notes, and thus, larger variability in examination methods, skills, and recording of findings. There were several individuals performing the palpation, some veterinarians, and some physiotherapists. This further increased the possibility of variation in the palpation technique, interpretation, and documentation. This, in addition to the lack of standardized palpation protocols is a limitation to our study, and may have had impact to our results, increasing type two bias error. A painful reaction to palpation in this study might also arise from overriding dorsal spinous processes or other tissues in the back. Radiography was not used in all of the horses’ back examinations, so other pathologies like overriding spinous processes were not excluded. Also, information regarding overriding dorsal spinous processes may have been omitted in some of the patient’s records. Other clinical signs or final diagnosis of the horses was not included in our data. Final diagnosis was not clear in all cases and sometimes was missing altogether. Although it might have been interesting, it was not imperative to the comparison of palpation and ultrasound findings. These factors may have impacted our study, as for example, pain in palpation without ultrasonographic findings in the SSL may have related to overriding dorsal spinous processes, thus being a true finding – just not SSL related. Therefore, painful palpation findings from other structures in the back without ultrasonographic changes in the SSL may impact especially the specificity of pain-related palpation findings.

Another key limitation is the diagnostic technique. Despite ultrasonography being the most useful technique for diagnosing SSL lesions (3), there are limitations for using it as a “gold standard” method. There is great variability in the SSL appearance on ultrasound even in horses with no SSL lesion (5). In the withers region the ossification centres induce hyperechoic images and in the caudal thoracic region, there is more fibrous pattern and echogenicity (3). Vertical hypoechoic images arise due to different head position and SSL relaxation. The interspinous area can give a false impression of hypoechogenicity (3). In a study of ridden and unridden horses without clinical signs of back pain, there were at least two abnormal areas in the SSL on ultrasonography (6). Also, in our study 12.7% of the horses had no palpatory findings, although ultrasonographic changes were seen. The interobserver variation related to the use and interpretation of the ultrasonography also needs to be considered. Changes in echogenicity are subjective interpretations, and sensitivity and specificity may vary with the observers’ ultrasonographic skills (27). As there is variation in normal echogenic pattern in the SSL, exclusion of other back disease is important in diagnostics of SSL injury. Local anesthesia of the SSL could give a more accurate diagnosis, but diffusion to other surrounding structures is possible (5). Using histopathologic evaluation of the SSL as the gold standard diagnosis would bring more reliability to the study but also be more costly and invasive. Palpatory and ultrasonography changes in the SSL exist, but the significance of these lesions in equine back pathology is still controversial (5).

Our results demonstrate that palpation is a highly sensitive method for assessing SSL-related signs, especially useful for detecting swelling in the thoracic spine SSL area. Palpation as a sensitive method could indicate SSL injuries and further need for ultrasonographic diagnostics.

## Data Availability

The datasets presented in this article are not readily available because of it being clinical patient information. All relevant information is published as results in this article. Requests to access the datasets should be directed to heli.hyytiainen@helsinki.fi.
